# The Intrinsic Antiviral Defense to Incoming HSV-1 Genomes Includes Specific DNA Repair Proteins and Is Counteracted by the Viral Protein ICP0

**DOI:** 10.1371/journal.ppat.1002084

**Published:** 2011-06-16

**Authors:** Caroline E. Lilley, Mira S. Chaurushiya, Chris Boutell, Roger D. Everett, Matthew D. Weitzman

**Affiliations:** 1 Laboratory of Genetics, The Salk Institute for Biological Studies, La Jolla, California, United States of America; 2 Graduate Program, Division of Biology, University of California, San Diego, California, United States of America; 3 MRC-University of Glasgow Centre for Virus Research, Glasgow, Scotland, United Kingdom; Cornell University, United States of America

## Abstract

Cellular restriction factors responding to herpesvirus infection include the ND10 components PML, Sp100 and hDaxx. During the initial stages of HSV-1 infection, novel sub-nuclear structures containing these ND10 proteins form in association with incoming viral genomes. We report that several cellular DNA damage response proteins also relocate to sites associated with incoming viral genomes where they contribute to the cellular front line defense. We show that recruitment of DNA repair proteins to these sites is independent of ND10 components, and instead is coordinated by the cellular ubiquitin ligases RNF8 and RNF168. The viral protein ICP0 targets RNF8 and RNF168 for degradation, thereby preventing the deposition of repressive ubiquitin marks and counteracting this repair protein recruitment. This study highlights important parallels between recognition of cellular DNA damage and recognition of viral genomes, and adds RNF8 and RNF168 to the list of factors contributing to the intrinsic antiviral defense against herpesvirus infection.

## Author Summary

The cellular DNA damage response pathway monitors damage to genomic DNA. We investigated whether cellular DNA damage response proteins can also respond to incoming viral genetic material and how they impact virus growth. Using Herpes Simplex Virus type 1 (HSV-1), we present evidence that DNA repair proteins are activated at the earliest times post-infection, and that they physically accumulate at sites associated with incoming viral genomes. A subset of these DNA repair proteins deposit repressive ubiquitin marks, recruit other DNA repair proteins, and limit transcription from the viral genomes. We demonstrate that the virus overcomes this anti-viral defense by targeting key DNA repair proteins for degradation. Our study adds these DNA repair protein mediators to the list of intrinsic antiviral defense factors active against HSV-1, and demonstrates that many aspects of the cellular recognition of foreign DNA parallel the recognition and response to cellular damage.

## Introduction

Mammalian cells have evolved complex defenses to protect themselves from viral infections. Innate and adaptive immune responses are well-characterized, but resistance mediated by pre-existing cellular factors has recently emerged as another important arm of antiviral defense. In contrast to the canonical immune responses, which are slower acting and initiated by virus-induced signaling cascades, the pre-existing cellular factors are poised to protect the cell before the virus has even entered [1]. This mechanism of resistance is called intrinsic antiviral defense, and is characterized by the fact that the antiviral proteins are intracellular and constitutively expressed, and that the restrictive factors can be overcome by viral countermeasures. These intrinsic defense pathways provide a primary protective mechanism in the first cell infected in an immunologically naive host, making them an important front line of defense against viruses.

Herpes simplex virus type 1 (HSV-1) is a common human pathogen that causes life-long recurrent disease. Lytic HSV-1 infection is characterized by transcription in a temporal cascade of immediate-early (IE), early (E), and late (L) gene products. The immediate early (IE) genes create a favorable intracellular environment for the virus, and regulate the expression of the E and L genes. The IE protein ICP0 is one of the first viral proteins expressed during HSV-1 infection (reviewed in [2]). Although ICP0 is a not an essential viral protein, its deletion significantly impairs productive replication, especially at low multiplicity of infection (MOI) [3]–[6]. ICP0 is a RING finger E3 ubiquitin ligase that induces degradation of several cellular proteins including the catalytic subunit of DNA-dependent protein kinase (DNA-PKcs) [7], the cellular DNA damage ubiquitin ligases RNF8 and RNF168 [8], components of the nuclear domain structures known as ND10 (or PML nuclear bodies) [9], [10], and centromeric proteins [11]–[13].

Prototypic intrinsic antiviral defense proteins, such as APOBEC3 proteins, are known to be active against a variety of viruses [1]. However, to date, the only proteins demonstrated to mediate intrinsic defense against herpesviruses are all components of ND10. The first evidence that these proteins may mediate intrinsic immune defense against herpesviruses came from the observation that depletion of PML increased the plaque-forming efficiency of both human cytomegalovirus (HCMV) [14] and ICP0-null HSV-1 [15]. Similarly, it was found that the ND10 proteins hDaxx and ATRX induce a repressive viral chromatin structure on incoming HCMV genomes that is prevented by the viral tegument protein pp71 targeting hDaxx for degradation [16]–[21]. Depletion of either hDaxx or ATRX also improves the plaque-forming efficiency of ICP0-null HSV-1, providing further evidence that ND10 proteins have a general role in mediating intrinsic antiviral defense against herpesviruses [22]. In the case of HSV-1, the repressive ND10 proteins have been detected at sites juxtaposed to incoming viral genomes [22]–[24]. During wild-type HSV-1 infection ICP0 rapidly disperses these inhibitory proteins, ensuring that replication can proceed. In the absence of ICP0, the recruitment of the ND10 proteins into novel structures associated with the viral genomes is readily observable as a very early cellular response, detectable within the first 30 minutes of infection [25]. ICP0 has therefore emerged as one of the key viral counterattacks to the cellular attempt to limit the early stages of infection.

Cells have elaborate machinery in place to monitor damage to genomic DNA and ensure the fidelity of replication [26]. Recent work has demonstrated that the cellular DNA repair machinery can also recognize viral genetic material [27]. HSV-1 has a complex relationship with the DNA damage response, in that it appears to activate many components of the ATM-dependent arm of the signaling pathway, while inhibiting the DNA-PKcs- and ATR-dependent arms [7], [28]–[30]. During lytic infection, HSV-1 recruits several cellular DNA repair proteins into viral replication compartments where they enhance viral replication [28]–[31]. Despite global activation of the ATM-dependent signaling pathway, we recently reported that RNF8 and RNF168, which are key mediators in this pathway, are targeted for proteasome-mediated degradation by ICP0 [8].

During HSV-1 infection, the viral capsid docks at the nuclear pore and the linear viral genome is released into the nucleus [32]. In this study, we asked whether the cellular DNA repair machinery recognizes this incoming viral DNA, and we explored the significance of ICP0-mediated degradation of RNF8 and RNF168 for the virus. We report that cellular DNA repair proteins respond to incoming HSV-1 genomes and we identify RNF8 and RNF168 as novel components of the intrinsic antiviral defense against HSV-1.

## Results

### Accumulation of cellular DNA repair proteins in response to incoming HSV-1 genomes

**Figure 1:**
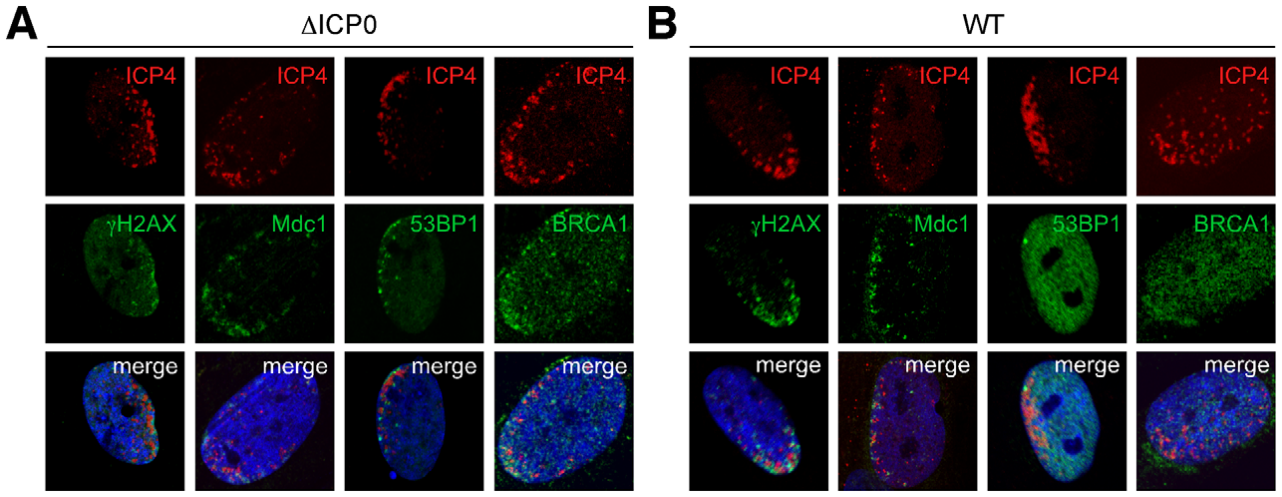
DNA repair proteins accumulate at sites associated with incoming HSV-1 genomes. (**A**) HFF cells were infected with ICP0-null HSV-1 at an MOI of 0.1 for 1 hr. Cells were fixed at 24 hpi, stained for ICP4, and localization of γH2AX, Mdc1, 53BP1 and BRCA1 was assessed in asymmetrically infected cells at edges of plaques. Nuclei were stained with DAPI as shown in the merged image. (**B**) HFF cells were infected with wild-type HSV-1 at an MOI of 0.001 for 1 hr and processed as in **A**.

In order to investigate effects of incoming HSV-1 genomes on localization of DNA damage proteins, we utilized a previously described assay to visualize nuclei at the earliest stages of infection [23], [24]. In this assay, cells are infected at low multiplicity so that directional viral spread through developing plaques can be analyzed. This directionality, combined with the fact that incoming viruses often congregate near the microtubule organizing center, means that nuclei of cells at the edge of plaques frequently display an asymmetric arc of incoming viral genomes [23], [24]. Human foreskin fibroblast (HFF) cells were infected at low MOI with wild-type or ICP0-null HSV-1, fixed 24 hours post-infection (hpi) and processed for immunofluorescence. Sites of incoming viral genomes were detected by staining with antiserum to the viral DNA binding protein, ICP4, which has been previously shown to co-localize with viral genomes in this assay [23], and the localization of certain cellular DNA repair proteins (Figure S1A) was assessed. In mock infected cells there was minimal γH2AX staining, and the damage checkpoint mediators Mdc1, 53BP1, and BRCA1 were localized in a diffuse nuclear pattern. In cells infected with ICP0-null virus, we detected that γH2AX, Mdc1, 53BP1, and BRCA1 accumulated in distinct asymmetric arcs in close proximity to incoming viral genomes (Figure 1A). In cells infected with wild-type virus, γH2AX and Mdc1 still re-localized to sites associated with viral DNA, but 53BP1 and BRCA1 remained diffusely nuclear (Figure 1B, Figure S1C). 53BP1 accumulated at sites associated with incoming ICP0-null viral genomes when high MOI infection was performed in the presence of α-amanitin, suggesting that viral transcription may not be essential (Figure S1B). These data indicate that redistribution of 53BP1 and BRCA1 in response to incoming viral genomes is an early response to HSV-1 infection that is inhibited by ICP0. We quantified the effect using 53BP1 and γH2AX as examples of DNA repair proteins that accumulated near incoming HSV-1 genomes. We observed that γH2AX accumulated near incoming HSV-1 genomes in over 80% of cells in both the presence and absence of ICP0 (Figure S1C). In contrast, while 53BP1 accumulated near incoming HSV-1 genomes in approximately 90% of cells infected with ICP0-null virus, this was reduced to approximately 25% of cells in the presence of ICP0 (Figure S1C).

### Sites of DNA repair protein accumulation are distinct from viral genomes and independent of ND10

**Figure 2:**
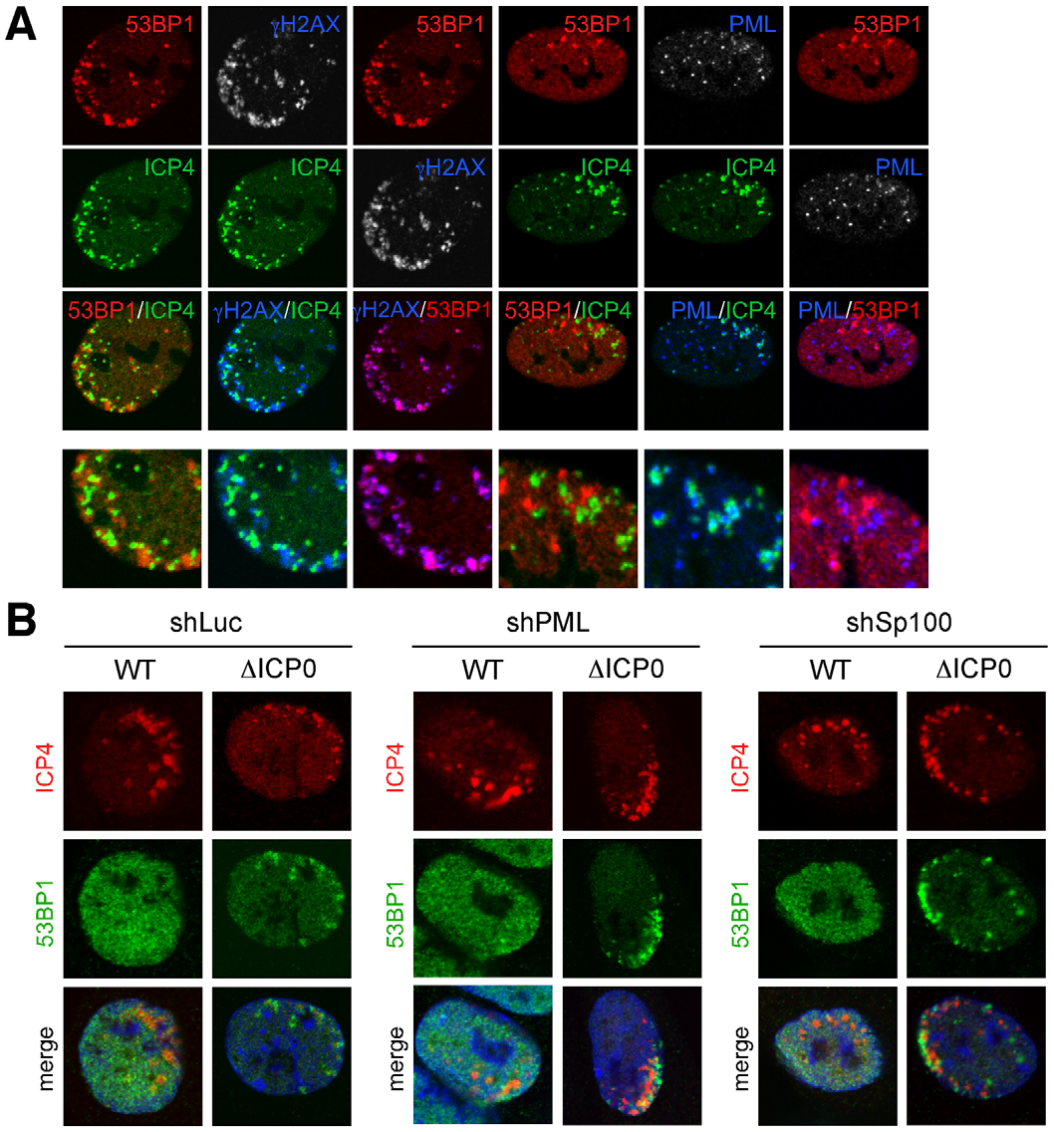
Sites of DNA repair protein accumulation are distinct from viral genomes, and independent of ND10 proteins. (**A**) HFF cells were infected with ICP0-null HSV-1 at an MOI of 0.1 for 1 hr. Cells were fixed at 24 hpi, stained for ICP4, and localization of γH2AX, 53BP1 and PML was assessed in asymmetrically infected cells. The lower panel represents enlarged images to show co-localization. (**B**) HepaRG cells depleted for PML, Sp100, or control shRNA treated cells were infected with wild-type virus at an MOI of 0.001 or ICP0-null HSV-1 at an MOI of 0.1 for 1 hr. Cells were fixed at 24 hpi, stained for ICP4, and localization of 53BP1 was assessed.

It has previously been reported that components of ND10, including hDaxx, PML, ATRX, and Sp100 accumulate at sites overlapping, but not precisely co-localizing with, incoming HSV-1 genomes [22]–[24]. We wished to determine if the virus-induced accumulation of DNA repair proteins we observed co-localized with either viral genomes or ND10 proteins. We found that while the γH2AX and 53BP1 staining co-localized, these DNA repair proteins did not co-localize with either ICP4 (representing viral genomes) or PML (representing ND10 proteins) (Figure 2A; see Figure S2A for the corresponding cytofluorograms). Despite this lack of co-localization, we observed a degree of overlap between the different structures. To analyze this, a Manders' overlap co-efficient [33] was determined for each image (Figure S2B). We observed that on average, approximately 50% of the PML signal overlapped with the ICP4 signal, whereas only 20% of the 53BP1 or γH2AX signal overlapped with the ICP4 signal. These data suggest that incoming viral genomes are more closely associated with ND10 proteins than DNA repair proteins, and that all three structures have subtly distinct sub-nuclear localizations.

Next, we investigated whether the accumulation of DNA repair proteins at sites of incoming viral genomes was dependent on major ND10 proteins. HepaRG cells depleted of PML or Sp100 [34] were infected with wild-type or ICP0-null HSV-1 and processed for immunofluoresence at 24 hpi. Infections in cells depleted for PML or Sp100 were indistinguishable from control cells with respect to γH2AX accumulation near incoming viral genomes in both the presence and absence of ICP0 (data not shown), while 53BP1 accumulated only in the absence of ICP0 (Figure 2B). Therefore, the recruitment of 53BP1 to incoming HSV-1 genomes and the ability of ICP0 to block this process are not dependent on either PML or Sp100. Taken together, these observations suggest that accumulation of ND10 proteins and DNA repair proteins are independent events occurring at distinct physical locations.

### Virus-induced repair foci are coordinated by RNF8 and RNF168

**Figure 3:**
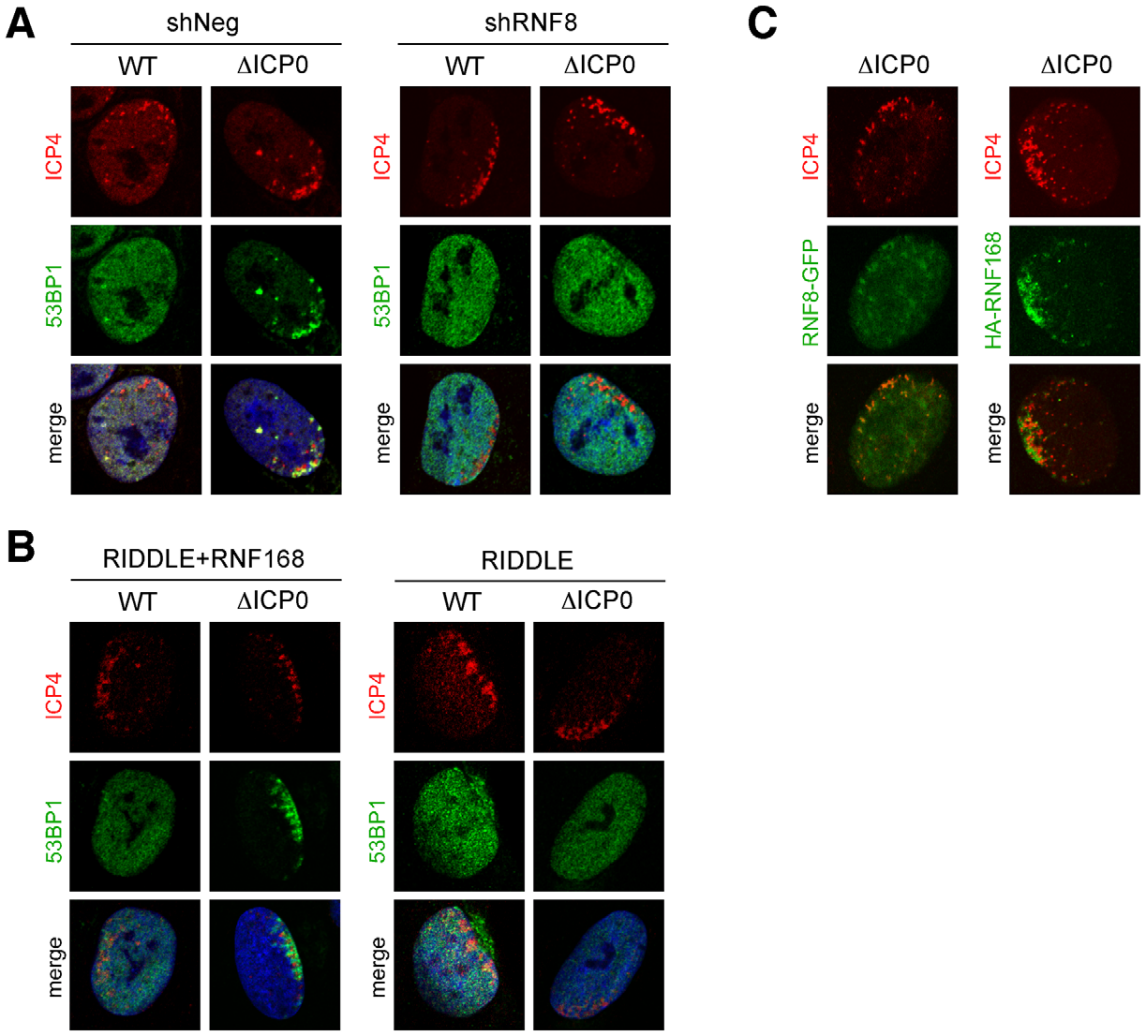
53BP1 accumulation at sites associated with incoming HSV-1 genomes is dependent on RNF8 and RNF168. (**A**) Control HepaRG cells or cells in which RNF8 had been depleted using shRNA were infected with wild-type virus at an MOI of 0.001 or ICP0-null HSV-1 at an MOI of 0.1 for 1 hr. Cells were fixed at 24 hpi, stained for ICP4, and 53BP1 localization was assessed in asymmetrically infected cells. (**B**) RIDDLE cells or RIDDLE cells complemented with HA-tagged RNF168 were infected and analyzed as in **A**. (**C**) HepaRG cells containing tet-inducible RNF8-GFP were induced with 0.1 µg/ml tet for 8 hrs and infected with ICP0-null HSV-1 at an MOI of 0.1. RIDDLE cells reconstituted with HA-tagged RNF168 were infected with ICP0-null HSV-1 at an MOI of 0.1. Cells were fixed at 16–24 hpi, stained for ICP4, and localization of RNF8-GFP or HA-RNF168 was assessed.

We recently reported that ICP0 expression leads to proteasome-mediated degradation of the cellular DNA repair proteins and histone ubiquitin ligases RNF8 and RNF168 [8]. We therefore investigated whether these proteins were responsible for coordinating the recruitment of 53BP1 to sites associated with ICP0-null viral genomes. We infected RNF8 depleted cells (Figure S3), or cells derived from a patient who has a biallelic mutation in RNF168 (RIDDLE cells, [35]) with wild-type or ICP0-null HSV-1 and assessed the recruitment of DNA repair proteins to incoming viral genomes (Figure 3A and B and Figure S4). During infection with wild-type virus, ICP0 expression prevented 53BP1 recruitment in the presence or absence of RNF8 and RNF168. However, in cells infected with ICP0-null virus, 53BP1 was not recruited to sites associated with incoming viral genomes in the absence of RNF8 or RNF168 (Figure 3A and B), despite the fact that γH2AX still accumulated (Figure S4). To determine if RNF8 and RNF168 themselves were recruited to sites associated with incoming viral genomes, we generated a cell line that could be induced to express GFP-tagged RNF8, or utilized RIDDLE cells complemented with a cDNA expressing HA-tagged RNF168. We observed that RNF168 clearly accumulated near incoming ICP0-null viral genomes (Figure 3C). Redistribution of RNF8 to the vicinity of HSV-1 genomes was also detectable, although this was weaker and more variable than recruitment of RNF168 (Figure 3C). Together, these data suggest that accumulation of RNF8 and RNF168 at sites associated with incoming viral genomes coordinates 53BP1 recruitment. This implies that the reason ICP0 targets RNF8 and RNF168 for degradation is to prevent recruitment of specific DNA repair factors to viral genomes, suggesting that this recruitment is detrimental to incoming virus during early stages of lytic infection.

### The requirements for 53BP1 recruitment parallel the cellular response to DNA damage

**Figure 4:**
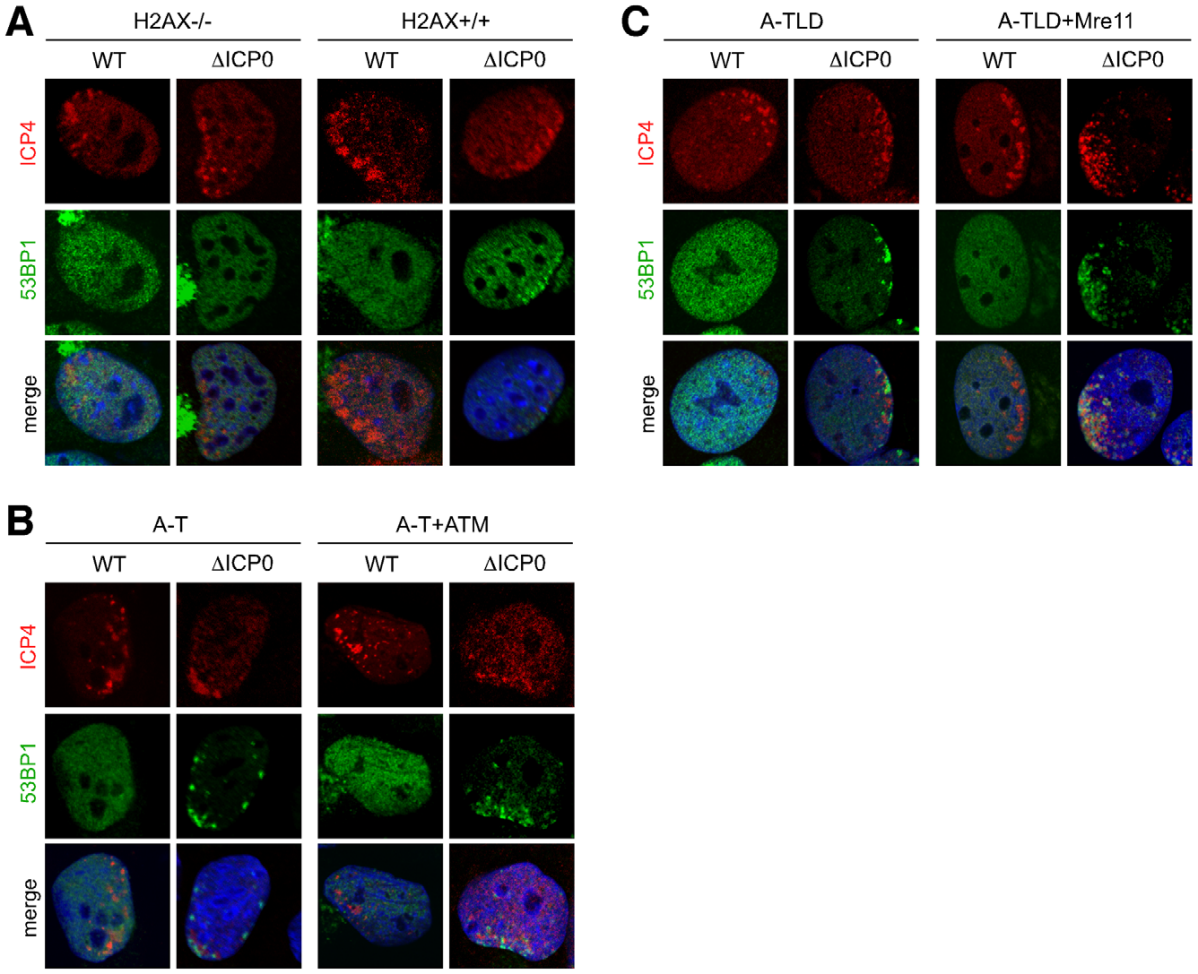
The requirements for 53BP1 recruitment parallel the cellular response to DNA damage. (**A**) H2AX-/- MEFs or matched control MEFs were infected with wild-type virus at an MOI of 0.001 or ICP0-null HSV-1 at an MOI of 0.1 for 1 hr. Cells were fixed at 24 hpi, stained for ICP4, and 53BP1 localization was assessed in asymmetrically infected cells. (**B**) A-T cells or cells in which ATM had been reconstituted were infected and processed as in **A**. (**C**) A-TLD-1 cells or A-TLD-1 cells in which Mre11 had been reconstituted were infected and processed as in **A**.

In uninfected mammalian cells, a tightly controlled hierarchy of events occurs following the induction of DNA double strand breaks [36], [37]. RNF8 and RNF168 coordinate the recruitment of 53BP1 to sites of cellular damage [38] and also to sites associated with incoming viral genomes. We therefore predicted that the latter process would be disrupted by depletion of factors upstream of RNF8 and RNF168 in the DNA damage response pathway. Phosphorylation of the histone variant H2AX is one of the first events to occur after induction of a double stranded DNA break [39], [40] and it is required for sustained accumulation of factors such as 53BP1 at damage sites [41], [42]. Phosphorylated H2AX binds MDC1, which in turn recruits RNF8 in a phosphorylation-dependent manner, and this interaction tethers 53BP1 and other downstream mediators at damage sites [38]. H2AX is therefore upstream of RNF8 and RNF168, and stable foci of 53BP1 do not form in H2AX-null cells. We infected cells from mice deleted for H2AX or matched control cells [43] with wild-type and ICP0-null HSV-1, and examined cells at the edge of developing plaques. As predicted, 53BP1 was recruited to ICP0-null viral genomes in wild-type mouse embryonic fibroblasts (MEFs), but did not accumulate during infection of cells lacking H2AX (Figure 4A).

ATM and the Mre11 complex are also upstream regulators of the cellular response to DNA damage. The Mre11 complex senses DNA double strand breaks and facilitates activation of ATM by recruiting it to the break sites [44]–[46]. However, despite this upstream role, 53BP1 still accumulates at sites of cellular DNA damage in cells deficient in Mre11 complex members or ATM [47]. We therefore assessed the requirement for ATM and Mre11 in coordinating the recruitment of 53BP1 to sites associated with ICP0-null viral genomes. We infected cells from patients with ataxia telangiectasia (A–T) and ataxia telangiectasia-like disorder (A-TLD) that lack functional ATM and Mre11 respectively, and compared them to matched controls in which ATM or Mre11 had been reconstituted. We observed that neither ATM (Figure 4B) or Mre11 (Figure 4C) were required for the accumulation of 53BP1 at sites associated with incoming ICP0-null viral genomes. These data demonstrate that H2AX, RNF8 and RNF168 are required for accumulation of 53BP1 at sites associated with incoming viral genomes, but ATM and Mre11 are not required. This hierarchy of signaling and recruitment events in response to viral genomes parallels the response to cellular DNA damage. Our data therefore suggest that the host cell recognizes either the incoming viral genomes themselves, or the resultant changes in local chromatin structure induced by incoming viral genomes, as DNA damage.

### Ubiquitination and SUMOylation mark sites associated with incoming viral genomes

**Figure 5:**
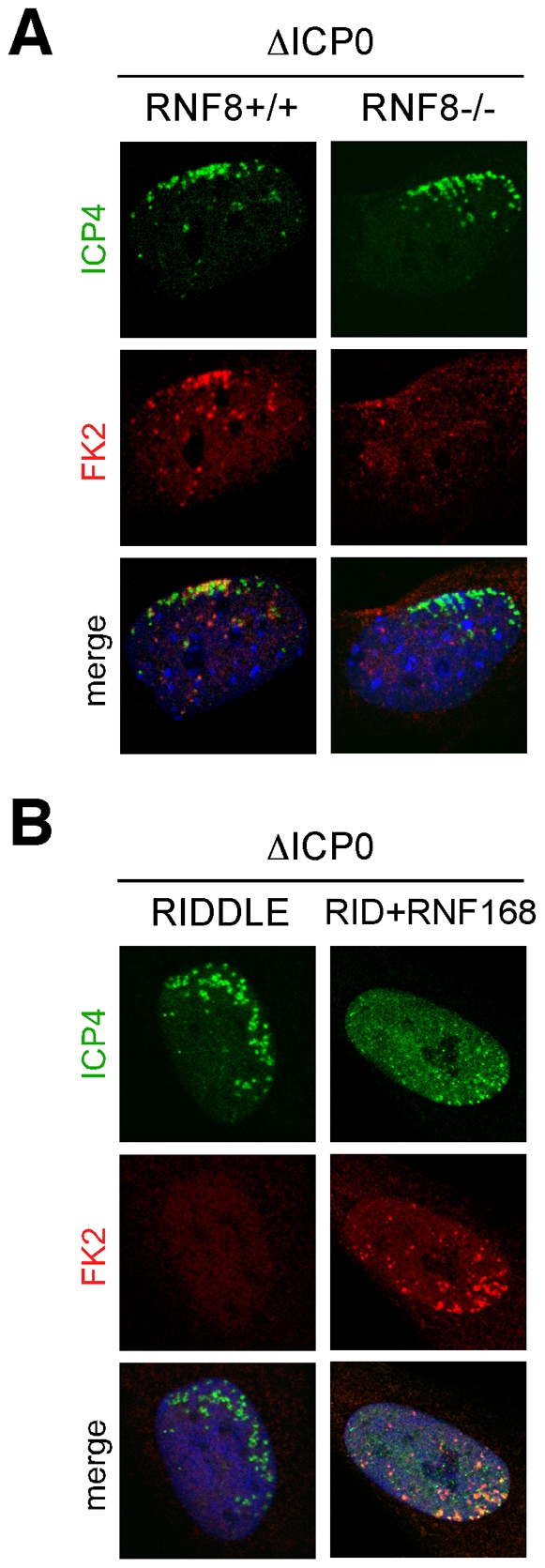
Conjugated ubiquitin accumulation at sites associated with incoming viral genomes is dependent on RNF8 and RNF168. (**A**) RNF8-/- MEFs or matched control MEFs were infected with ICP0-null HSV-1 at an MOI of 0.1 for 1 hr. Cells were fixed at 24 hpi, stained for ICP4, and localization of conjugated ubiquitin (FK2) was assessed in asymmetrically infected cells. (**B**) RIDDLE cells or RIDDLE cells expressing HA-tagged RNF168 were infected and analyzed as in **A**.

RNF8 and RNF168 are ubiquitin ligases for the histone H2A [35], [48]–[51], and we have previously reported that ICP0 expression leads to loss of uH2A, concomitant with the degradation of these two ligases [8]. We therefore examined ubiquitin conjugation at the sites associated with incoming ICP0-null viral genomes. We infected RNF8-null MEFs, RIDDLE cells, and matched controls, with ICP0-null virus and examined conjugated ubiquitin staining (FK2) at sites associated with incoming viral genomes at 24 hpi (Figure 5A). Asymmetric FK2 staining was detectable only in cells expressing RNF8 and RNF168, suggesting that this represents uH2A, which we also detected associated with incoming ICP0-null viral genomes (Figure S5A). The FK2 signal co-localized with 53BP1, but not PML, at sites associated with incoming viral genomes, suggesting that conjugated ubiquitin was a marker for sites of DNA repair protein accumulation rather than sites of ND10 protein accumulation (Figure S5B).

**Figure 6:**
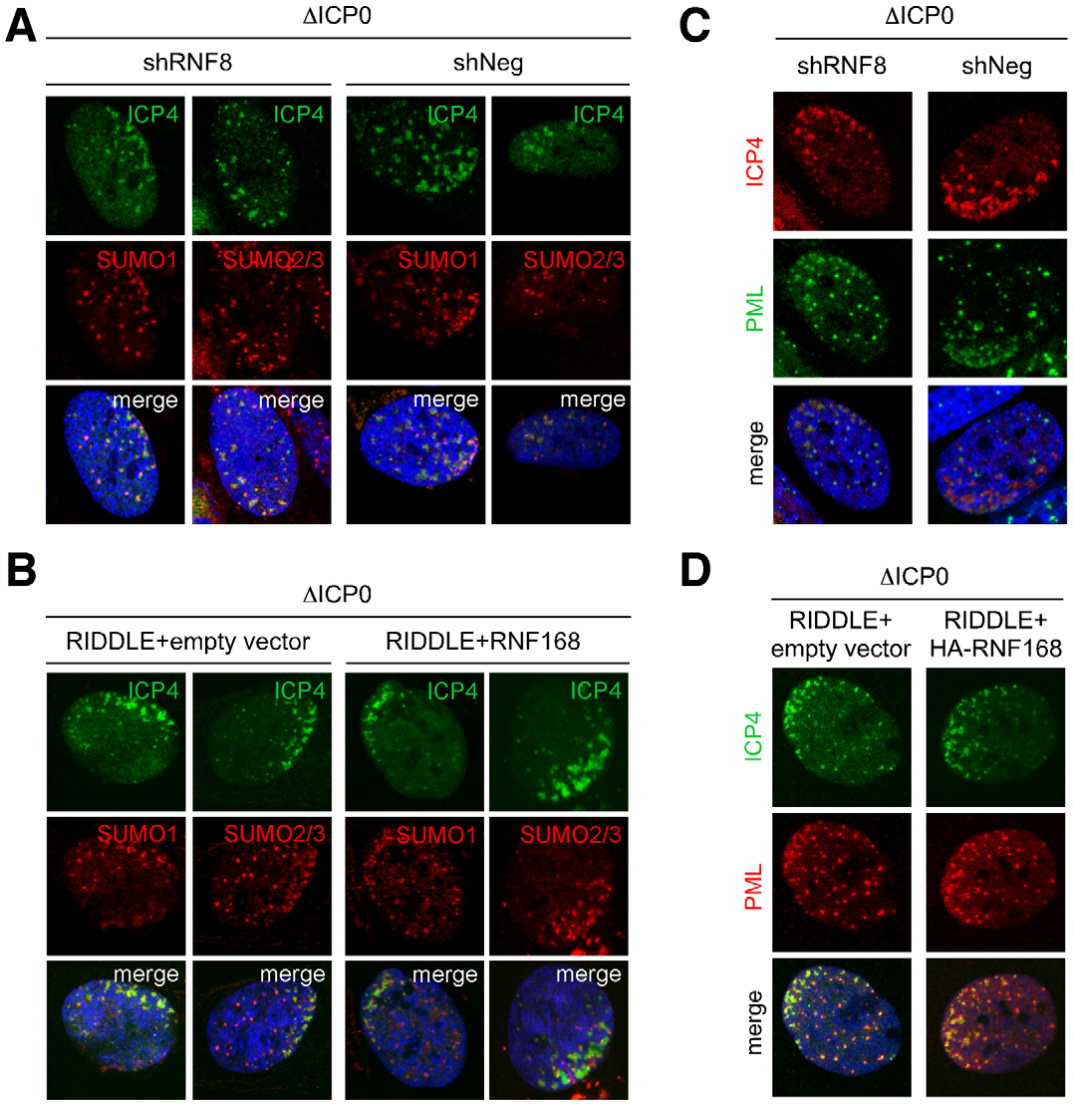
SUMO accumulation at sites associated with incoming viral genomes is not dependent on RNF8 or RNF168. (**A**) Control cells or cells in which RNF8 had been depleted using shRNA were infected with ICP0-null HSV-1 at an MOI of 0.1 for 1 hr. Cells were fixed at 24 hpi, stained for ICP4, and localization of SUMO1 or SUMO2/3 was assessed in asymmetrically infected cells. (**B**) RIDDLE cells or RIDDLE cells with HA-tagged RNF168 reconstituted were infected and analyzed as in **A**. (**C**) Control HFF cells or cells in which RNF8 had been depleted using shRNA were infected as in A and assessed for localization of PML. (**D**) RIDDLE cells or RIDDLE cells with HA-tagged RNF168 reconstituted were infected and analyzed as in **C**.

SUMO modification has also recently emerged as an important regulator of cellular DNA damage signaling [52], [53] and SUMO conjugates have been detected at sites associated with incoming ICP0-null genomes (Cuchet-Lourenco, Boutell and Everett, unpublished observations). In the case of cellular DNA double strand breaks, SUMO1 and SUMO2/3 recruitment is dependent on RNF8 and RNF168 [52]. We therefore determined whether SUMO recruitment to sites associated with incoming ICP0-null genomes was also dependent on RNF8 and RNF168. We infected cells depleted for RNF8 or lacking functional RNF168, and their matched controls, with ICP0-null virus and analyzed cells at the edges of developing plaques for asymmetric accumulations of SUMO. Both SUMO1 and SUMO2/3 were recruited to sites associated with incoming ICP0-null genomes even in the absence of RNF8 or RNF168 (Figure 6A and B). ND10 proteins are heavily SUMOylated, and SUMO modified forms of PML and Sp100 are known to be targets of ICP0 [15]. We therefore speculate that at least some of the SUMO conjugates we detected in the absence of RNF8 and RNF168 may represent sites of ND10 protein accumulation rather than DNA repair proteins, an idea supported by the observation that PML is still recruited to these sites in cells depleted for RNF8 and RNF168 (Figure 6C and D). Together, these data show that recruitment of ND10 components and DNA repair proteins are independent events, sharing the common themes of being disrupted by ICP0 and likely being coordinated by SUMO modification events.

### Recruited factors can mediate repression of incoming genomes: intrinsic antiviral defense

Accumulation of cellular factors at sites associated with incoming HSV-1 genomes has been strongly linked to restricting the invading virus [22], [34]. We therefore wished to determine the biological significance of the accumulation of specific DNA repair proteins at sites associated with incoming viral genomes.

**Figure 7:**
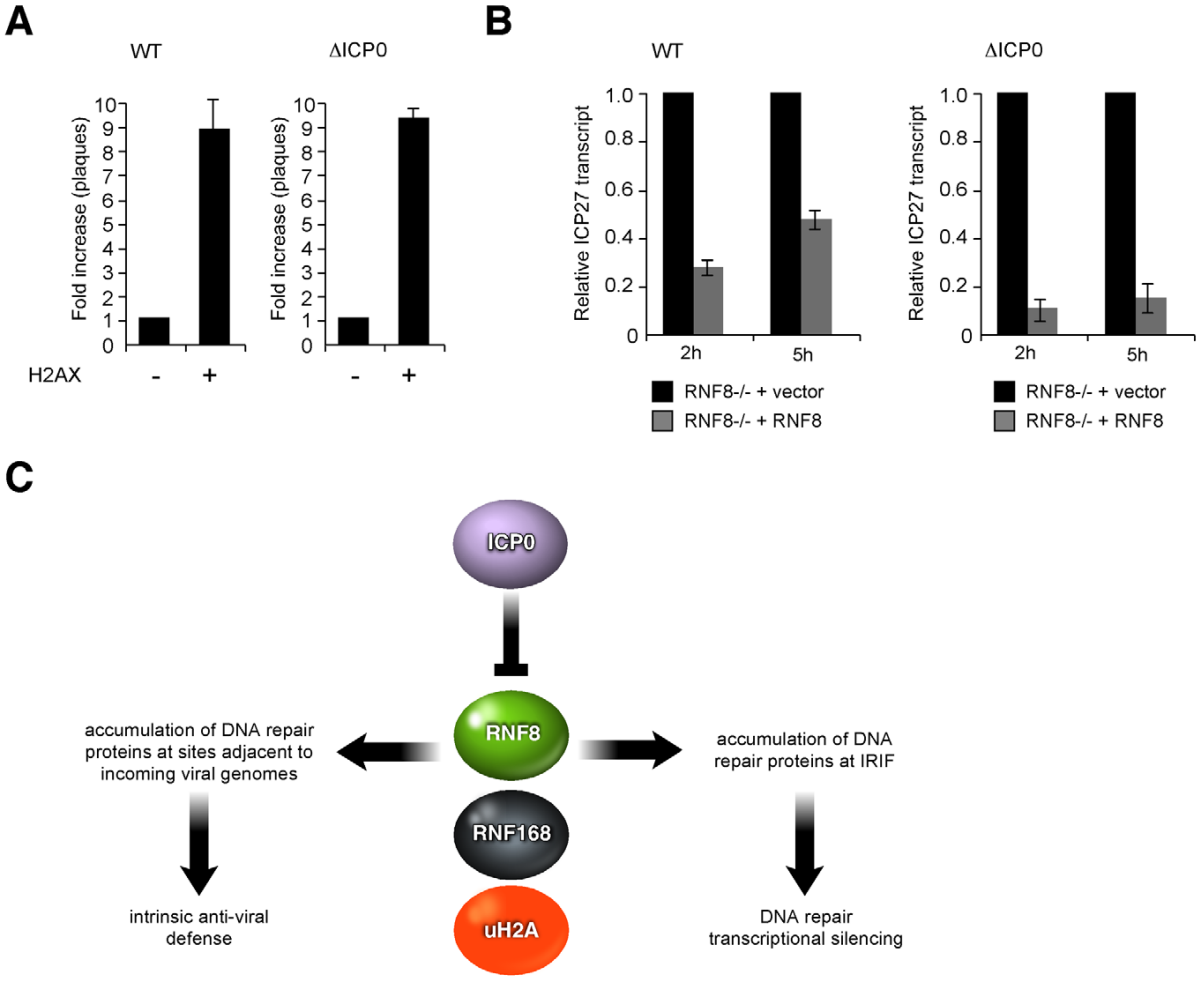
H2AX is beneficial for HSV-1 replication while RNF8 represses viral genomes. (**A**) H2AX-/- MEFs or matched controls were infected with wild-type or ICP0-null virus. Relative probabilities of plaque formation were calculated by counting the numbers of plaques on the different cell lines at each separate dilution of virus. Infections were carried out at least in duplicate and the experiment was repeated four times. Data are represented as mean +/- SEM. (**B**) RNF8-/- MEFs expressing RNF8 or empty vector were infected with WT or ICP0-null virus at an MOI of 0.01. ICP27 transcripts were detected at 2 or 5 hpi and normalized to a cellular control. Data were analyzed by comparing transcription in the presence of RNF8 to transcription in the absence of RNF8 and therefore represent fold repression by RNF8 for each virus. Experiments were performed in duplicate and averaged. Results are representative of three independent experiments and the error is one standard deviation of the duplicate samples. (**C**) Model showing the parallels between sites of cellular DNA damage and sites associated with incoming HSV-1 genomes and the role of central role of ICP0, RNF8 and RNF168 in disrupting both structures.

First, we assessed the ability of wild-type or ICP0-null virus to form plaques on cells deficient for H2AX or matched control cells expressing wild-type H2AX. We observed that both wild-type and ICP0-null HSV-1 were approximately 10-fold more likely to form plaques in the presence of H2AX (Figure 7A). This is similar to our previous data demonstrating that certain DNA repair proteins, such as ATM and Mre11, are beneficial for HSV-1 replication [28], possibly via processing of intermediates generated during viral replication/recombination [54]. Even though γH2AX is excluded from viral replication compartments [55], this histone variant is one of the master regulators of DNA damage signaling, and it is likely that H2AX phosphorylation is required to activate or recruit specific downstream proteins required during viral replication.

Our FK2 data (Figure 5) suggested that ubiquitination events at sites associated with incoming viral genomes are regulated by RNF8 and RNF168. Since uH2A has well-characterized roles in silencing [56]–[58] and ICP0 is a known transcriptional activator, we hypothesized that one reason for ICP0 to target RNF8 and RNF168 is to limit transcriptional repression of incoming viral genomes. To test this hypothesis, we compared the transcriptional competence of viral genomes in the presence and absence of RNF8 (Figure 7B). Cells from RNF8 null mice transduced with empty retrovirus or retrovirus expressing human WT RNF8 [8] were infected with wild-type or ICP0-null HSV-1 and harvested at 2 and 5 hpi. RNA was isolated and reverse transcribed, and qPCR was performed to detect ICP27 transcripts as a marker of viral transcription. We confirmed that input DNA was similar in all infections (data not shown) and analyzed the data by comparing transcription in the presence of RNF8 to transcription in the absence of RNF8 (Figure 7B; see Figure S6A for transcript levels across all samples). We observed that a) both viruses were transcriptionally repressed by RNF8, b) this repression was more significant in the absence of ICP0 and c) RNF8-mediated repression decreased over time during wild-type but not ICP0-null virus infection, presumably as a consequence of RNF8 degradation (Figure 7B). These data indicate that RNF8 is transcriptionally repressive to HSV-1 genomes and explains why HSV-1 forms plaques less efficiently in the presence of RNF8 [8] and/or RNF168 (Figure S6B and C).

## Discussion

In this study we discovered that RNF8 and RNF168 coordinate a repressive barrier to incoming HSV-1 genomes, and that ICP0 targets these cellular ubiquitin ligases to overcome this host antiviral effect. We describe structures marked by DNA repair proteins and conjugated ubiquitin that form *de novo* in response to incoming viral genomes. These DNA repair structures are associated with, but are independent of, similar ND10-like structures that also form near incoming viral genomes.

Our studies highlight the complexity of the interface between HSV-1 and the cellular DNA damage response. Previous work demonstrated that certain recombination and repair proteins, such as Mre11, ATM, ATR/ATRIP, and WRN are beneficial for HSV-1 replication [28], [29], [31]. Here we show that H2AX is also required for optimal replication of HSV-1, as previously suggested [59]. In contrast, the NHEJ proteins, DNA-PKcs and Ku70, have been reported to be detrimental to HSV-1 replication [7], [31]. We found that RNF8 and RNF168 also inhibit replication, likely by creating a repressive environment at the nuclear sites of incoming viral genomes. Together, these observations suggest that HSV-1 temporally dissects the DNA repair pathway; this ensures that repressive proteins are degraded, while repair proteins required to coordinate signaling and facilitate replication or processing of viral genomes are retained.

Although accumulation of many cellular factors has been strongly linked to restricting the incoming viral genomes [15], [22], [34], recruitment does not necessarily always correlate with repression. For example, some PML isoforms accumulate at sites associated with incoming HSV-1 genomes but do not inhibit the plaque-forming ability of ICP0-null virus (Cuchet-Lourenco, Boutell and Everett, unpublished observations). Similarly, we observe γH2AX accumulation at sites associated with incoming viral genomes, but find that H2AX is required for optimal HSV-1 replication. In contrast, proteins involved in intrinsic antiviral defense are not only recruited to incoming genomes, but limit viral progression, and are therefore inactivated by the virus during the earliest stages of infection. Our data identify RNF8 and RNF168 as new members of the host cell antiviral arsenal against incoming HSV-1.

When HSV-1 genomes enter the nucleus, they do so as naked DNA. However, the cell responds by depositing repressive chromatin marks on the incoming nucleic acid [60]–[64]. In turn, the virus recruits modification complexes containing histone demethylases and methyltransferases, and installs positive marks to facilitate IE transcription [65]. These demethylases may act in concert with histone deacetylases, such as HDAC1, which bind the transcriptionally repressive coREST/REST complex in the absence of ICP0 [66], [67]. We observed γH2AX and uH2A in association with sites of incoming viral genomes at the earliest detectable stages of infection, suggesting that these post-translational histone modifications are a very early response to incoming viral DNA. Our co-localization studies raise the possibility that these modified histones may be deposited on the displaced host chromatin around the incoming viral genomes.

Our data highlight the emerging parallels between cellular recognition of viral DNA and the cellular response to DNA damage. In both cases, γH2AX is activated, Mdc1 accumulates, and downstream repair factors such as 53BP1 are recruited. Furthermore, both processes are coordinated by the ubiquitin ligases RNF8 and RNF168, and ICP0 is thus able to disrupt both by inducing the degradation of these cellular proteins (Figure 7C). However, in contrast to the situation at sites of cellular DNA damage, we observed that SUMO conjugates still accumulate near incoming viral genomes even in the absence of RNF8 or RNF168. This accumulation likely reflects SUMO modification of ND10 components, which we show are still recruited in the absence of RNF8 or RNF168. Recent work has demonstrated that the SIMs of PML, hDaxx and Sp100 are essential for their recruitment to virus-induced foci (Cuchet-Lourenco, Boutell and Everett, unpublished observations) raising the possibility that these ND10 components are recruited in response to upstream SUMO-dependent events at these sites. RNF168 is known to contain SIMs and its recruitment to sites of cellular damage depends on the SUMO ligase PIAS4 [52]. It will therefore be interesting to determine whether the SIMs in RNF168 are required for its accumulation near incoming viral genomes, and whether disrupting the SUMO pathway can abrogate accumulation of both ND10 proteins and DNA repair proteins at these sites. Conversely, it will be interesting to see if the accumulation of ND10 components at sites of cellular DNA damage [68], [69] is SUMO-dependent and whether this still occurs in the absence of RNF8 and RNF168.

It has recently been shown that sites of cellular DNA damage are characterized by transcriptional repression [70]. The parallels we have uncovered between recruitment of DNA repair proteins to sites of cellular DNA damage and to incoming viral genomes raise the possibility that silencing is a defining characteristic of both sites. We propose that the accumulation of SUMO conjugates, ND10 components and DNA repair proteins are hallmarks of a repressive cellular response to both damaged and foreign DNA.

## Materials and Methods

### Cell lines

Vero and U20S cells were purchased from the American Tissue Culture Collection. MEFs from RNF8-/- knockout mice and matched wild-type controls were obtained from Razq Hakem [71] or Junjie Chen [72] and for some experiments RNF8-/- MEFs were complemented with human RNF8 [8] were used. Human foreskin fibroblasts (HFFs), obtained from the University of California San Diego Medical Center, were kindly provided by Debbie Spector. Cells were maintained in Dulbecco modified Eagle's medium (DMEM) containing 100 U/ml of penicillin and 100 µg/ml of streptomycin, supplemented with 10% fetal bovine serum (FBS) and selection antibiotics as appropriate. Cells were grown at 37°C in a humidified atmosphere containing 5% CO_2_. HepaRG hepatocyte cells [73] were grown in William's medium E supplemented with 2 mM glutamine, 5 µg/ml insulin, and 0.5 µM hydrocortisone. H2AX-/- MEFs were obtained from Andre Nussenzweig [43]. A-T cells (AT22IJE-T) and matched ATM put-back cells were obtained from Yosef Shiloh [74]. A-TLD-1 cells and matched cells with Mre11 reconstituted were described previously [75]. The inducible RNF8-GFP cell line was constructed by cloning RNF8 into the previously described tet-inducible pLKO based expression system [76]. Cells were induced with 0.1 µg/ml tetracycline for 8 hrs.

### shRNA constructs

shRNA targeting RNF8 was 5′ ACATGAAGCCGTTATGAAT 3′ as previously described [49]. This sequence was incorporated into a pLKO based (for HepaRG cells) or GFP-tagged HIV vector plasmids [77].

### Viruses and infections

Parental virus HSV-1 strain was 17 syn+ and the matched ICP0 deletion mutant was *dl*1403 [5]. Viruses were grown in Vero cells and titered in U2OS cells, in which ICP0 is not required for efficient plaque formation. Infections were performed on monolayers of cells in DMEM with 0% FBS. After 1 hr at 37°C, virus was removed and media containing 10% FBS was added. For plaque edge experiments, this media was supplemented with 1% human serum to limit spread of the virus. For plaque assays, 24 well dishes were infected with three-fold dilutions of wild-type or ICP0-null HSV-1. After adsorption, the cells were overlaid with medium containing 10% FBS and 1% human serum. Plaques were stained with crystal violet 24–36 h post-infection. Pseudotyped lentiviral stocks were generated by transfecting 293T cells with the appropriate vector plasmid and pVSV-G, pRev and pMDL plasmids as previously described [77].

### Antibodies

Primary antibodies were purchased from Bethyl (PML), Abcam (SUMO1 and SUMO2/3), Rockland (ATM S1981-P), Santa Cruz (BRCA1, 53BP1), Millipore (H2AX S139, FK2, H2A, uH2A), Calbiochem (BRCA1), Research Diagnostics Inc. (GAPDH), Covance (HA), Transduction Laboratories (DNA-PKcs), and Sigma (FLAG). Rabbit antisera to Mdc1 was from J. Chen. The 58S monoclonal antibody to ICP4 was generated from an ATCC hybridoma cell line [78]. All secondary antibodies were from Jackson Laboratories or Invitrogen.

### Immunoblotting and immunofluorescence

For immunoblotting, lysates prepared by standard methods. For immunofluorescence, cells were fixed with 4% paraformaldehyde for 15 min and extracted with 0.5% Triton X-100 in PBS for 10 min. For certain antibodies, cells were pre-treated with 0.5% Triton X-100 in PBS for 10 min prior to fixation. Nuclei were visualized by staining with DAPI. Images were acquired using a Leica TCS SP2 confocal microscope.

### qPCR

2X10^6^ cells were infected with WT or ICP0-null virus at an MOI of 0.01 and harvested at 2 and 5 hpi. 75% of the cell pellet was used for RNA extraction and 25% for DNA purification. 1 µg RNA was reverse transcribed using SuperScriptIII RT (Invitrogen) and oligo dT in a 20 µl reaction. qPCR was run in triplicate with 3 µl cDNA or 100 ng genomic DNA using SYBR Green PCR master mix (ABI) on an ABI 7900HT system. ICP27 transcript was detected using primers GCATCCTTCGTGTTTGTCATT (F) and GCATCTTCTCTCCGACCCCG (R) [65] and normalized to endogenous RPLPO transcript detected using primers CTGGAAGTCCAACTACTTCC (F) and TGCTGCATCTGCTTGGAGCC (R).

## Supporting Information

Figure S1 Overview of the cellular response to DNA damage, requirements for recruitment of 53BP1 to sites associated with ICP0-null HSV-1 genomes and quantification of the recruitment phenotype. **(A)** Simplified overview of the cellular response to DNA damage, indicating the positions of key proteins used in our studies **(B)** HFF cells were treated with 50 µg/ml α-amanitin for 1 hr prior to infection with ICP0-null HSV-1 at an MOI of 10 for 4 hrs in the presence of 50 µg/ml α-amanitin. Cells were fixed and stained for ICP4 (to confirm that transcription was blocked), and localization of γH2AX and 53BP1 was assessed. Nuclei were stained with DAPI as shown in the merged image. **(C)** HFF cells were infected with wild-type or ICP0-null HSV-1 at an MOI of 0.001 or 0.1, respectively for 1 hr, and virus was replaced with media containing 1% human serum to limit viral spread. Cells were fixed at 24 hpi, stained for ICP4, and localization of γH2AX (left graph) or 53BP1 (right graph) was assessed in 100 asymmetrically infected cells at edges of plaques.
(TIF)

Figure S2 Sites of DNA repair protein accumulation are distinct from viral genomes and de novo ND10 structures. **(A)** HFF cells were infected with ICP0-null HSV-1 at an MOI of 0.1 for 1 hr, and virus was replaced with media containing 1% human serum. Cells were fixed at 24 hpi, and the correlation between pixels positive for 53BP1 and pixels positive for γH2AX (top panel), 53BP1 and ICP4 (middle panel) or γH2AX and ICP4 (bottom panel) was assessed in one representative asymmetrically infected triple-labeled cell. **(B)** Cells were infected as in **A**, and stained to assess ICP4, 53BP1, and PML localization. The Manders' overlap co-efficient was determined for ICP4 and 53BP1 overlap compared to ICP4 and PML overlap in one representative asymmetrically infected triple-labeled cell.
(TIF)

Figure S3 Characterization of RNF8-depleted cells. **(A)** HepaRG cells were infected with lentivirus expressing shRNA specific to RNF8 as described in the methods section. Cells were isolated under puromycin selection and levels of RNF8 were assessed by western blot. **(B)** HepaRG shRNF8 cells were irradiated with 10 Gy γIR and the localization of 53BP1 was assessed by immunofluorescence.
(TIF)

Figure S4 γH2AX accumulation at sites associated with incoming HSV-1 genomes is not dependent on RNF8 or RNF168. **(A)** Control HepaRG cells or cells in which RNF8 had been depleted using shRNA were infected with wild-type virus at an MOI of 0.001 or ICP0-null HSV-1 at an MOI of 0.1 for 1 hr, and virus was replaced with media containing 1% human serum. Cells were fixed at 24 hpi, stained for ICP4, and γH2AX localization was assessed in asymmetrically infected cells at edges of plaques. **(B)** RIDDLE cells or RIDDLE cells with HA-tagged RNF168 reconstituted were infected and analyzed as in **A**.
(TIF)

Figure S5 uH2A and conjugated ubiquitin accumulation at sites of incoming ICP0-null HSV-1 genomes. **(A)** HFF cells were infected with wild-type or ICP0-null HSV-1 at an MOI of 0.001 or 0.1 respectively for 1 hr, and virus was replaced with media containing 1% human serum. Cells were pre-extracted before fixation at 24 hpi, and localization of uH2A was assessed in asymmetrically infected cells at edges of plaques. **(B)** HFF cells were infected with ICP0-null HSV-1 at an MOI 0.1 for 1 hr, and virus was replaced with media containing 1% human serum. Cells were fixed at 24 hpi, and stained for ICP4, PML, and FK2 (conjugated ubiquitin) or ICP4, 53BP1, and FK2. The correlation between pixels positive for 53BP1 and FK2 (top panel), or PML and ICP4 (bottom panel) was assessed in the images shown.
(TIF)

Figure S6 RNF8 represses viral genomes and is part of the intrinsic anti-viral defense. **(A)** RNF8-/- MEFs transduced with control retrovirus or retrovirus expressing RNF8 were infected with WT or ICP0 null virus at an MOI of 0.01. ICP27 transcripts were detected at 2 or 5 hrs post infection and normalized to a cellular control. In both cell lines, ICP0 null virus was less transcriptionally competent than wild-type, in keeping with the known phenotype of this mutant virus. Experiments were performed in duplicate and averaged. Results are representative of three independent experiments and error is one standard deviation of the duplicate samples. **(B)** RIDDLE cells complemented either with empty vector or RNF168 were treated with lentivirus expressing either control shRNA or shRNA targeting RNF8. Levels of RNF8 and uH2A were assessed by western blot. **(C)** Cells depleted for RNF168 and/or RNF8 were infected with wild-type or ICP0-null virus. Relative probabilities of plaque formation were calculated by comparing the numbers of plaques on the different cell lines at each separate dilution of virus.
(TIF)

## References

[1] Bieniasz PD (2004). Intrinsic immunity: a front-line defense against viral attack.. Nat Immunol.

[2] Everett RD (2000). ICP0, a regulator of herpes simplex virus during lytic and latent infection.. Bioessays.

[3] Cai W, Schaffer PA (1992). Herpes simplex virus type 1 ICP0 regulates expression of immediate-early, early, and late genes in productively infected cells.. J Virol.

[4] Chen J, Silverstein S (1992). Herpes simplex viruses with mutations in the gene encoding ICP0 are defective in gene expression.. J Virol.

[5] Stow ND, Stow EC (1986). Isolation and characterization of a herpes simplex virus type 1 mutant containing a deletion within the gene encoding the immediate early polypeptide Vmw110.. J Gen Virol.

[6] Everett RD, Boutell C, Orr A (2004). Phenotype of a herpes simplex virus type 1 mutant that fails to express immediate-early regulatory protein ICP0.. J Virol.

[7] Parkinson J, Lees-Miller SP, Everett RD (1999). Herpes simplex virus type 1 immediate-early protein vmw110 induces the proteasome-dependent degradation of the catalytic subunit of DNA-dependent protein kinase.. J Virol.

[8] Lilley CE, Chaurushiya MS, Boutell C, Landry S, Suh J (2010). A viral E3 ligase targets RNF8 and RNF168 to control histone ubiquitination and DNA damage responses.. EMBO J.

[9] Everett RD, Freemont P, Saitoh H, Dasso M, Orr A (1998). The disruption of ND10 during herpes simplex virus infection correlates with the Vmw110- and proteasome-dependent loss of several PML isoforms.. J Virol.

[10] Gu H, Roizman B (2003). The degradation of promyelocytic leukemia and Sp100 proteins by herpes simplex virus 1 is mediated by the ubiquitin-conjugating enzyme UbcH5a.. Proc Natl Acad Sci U S A.

[11] Everett RD, Earnshaw WC, Findlay J, Lomonte P (1999). Specific destruction of kinetochore protein CENP-C and disruption of cell division by herpes simplex virus immediate-early protein Vmw110.. EMBO J.

[12] Lomonte P, Sullivan KF, Everett RD (2001). Degradation of nucleosome-associated centromeric histone H3-like protein CENP-A induced by herpes simplex virus type 1 protein ICP0.. J Biol Chem.

[13] Lomonte P, Morency E (2007). Centromeric protein CENP-B proteasomal degradation induced by the viral protein ICP0.. FEBS Lett.

[14] Tavalai N, Papior P, Rechter S, Leis M, Stamminger T (2006). Evidence for a role of the cellular ND10 protein PML in mediating intrinsic immunity against human cytomegalovirus infections.. J Virol.

[15] Everett RD, Rechter S, Papior P, Tavalai N, Stamminger T (2006). PML contributes to a cellular mechanism of repression of herpes simplex virus type 1 infection that is inactivated by ICP0.. J Virol.

[16] Woodhall DL, Groves IJ, Reeves MB, Wilkinson G, Sinclair JH (2006). Human Daxx-mediated repression of human cytomegalovirus gene expression correlates with a repressive chromatin structure around the major immediate early promoter.. J Biol Chem.

[17] Saffert RT, Kalejta RF (2006). Inactivating a cellular intrinsic immune defense mediated by Daxx is the mechanism through which the human cytomegalovirus pp71 protein stimulates viral immediate-early gene expression.. J Virol.

[18] Preston CM, Nicholl MJ (2006). Role of the cellular protein hDaxx in human cytomegalovirus immediate-early gene expression.. J Gen Virol.

[19] Lukashchuk V, McFarlane S, Everett RD, Preston CM (2008). Human cytomegalovirus protein pp71 displaces the chromatin-associated factor ATRX from nuclear domain 10 at early stages of infection.. J Virol.

[20] Cantrell SR, Bresnahan WA (2006). Human cytomegalovirus (HCMV) UL82 gene product (pp71) relieves hDaxx-mediated repression of HCMV replication.. J Virol.

[21] Tavalai N, Papior P, Rechter S, Stamminger T (2008). Nuclear domain 10 components promyelocytic leukemia protein and hDaxx independently contribute to an intrinsic antiviral defense against human cytomegalovirus infection.. J Virol.

[22] Lukashchuk V, Everett RD (2010). Regulation of ICP0-null mutant herpes simplex virus type 1 infection by ND10 components ATRX and hDaxx.. J Virol.

[23] Everett RD, Murray J (2005). ND10 components relocate to sites associated with herpes simplex virus type 1 nucleoprotein complexes during virus infection.. J Virol.

[24] Everett RD, Sourvinos G, Leiper C, Clements JB, Orr A (2004). Formation of nuclear foci of the herpes simplex virus type 1 regulatory protein ICP4 at early times of infection: localization, dynamics, recruitment of ICP27, and evidence for the de novo induction of ND10-like complexes.. J Virol.

[25] Everett RD, Murray J, Orr A, Preston CM (2007). Herpes simplex virus type 1 genomes are associated with ND10 nuclear substructures in quiescently infected human fibroblasts.. J Virol.

[26] Jackson SP, Bartek J (2009). The DNA-damage response in human biology and disease.. Nature.

[27] Weitzman MD, Lilley CE, Chaurushiya MS (2010). Genomes in conflict: maintaining genome integrity during virus infection.. Annu Rev Microbiol.

[28] Lilley CE, Carson CT, Muotri AR, Gage FH, Weitzman MD (2005). DNA repair proteins affect the lifecycle of herpes simplex virus 1.. Proc Natl Acad Sci U S A.

[29] Mohni KN, Livingston CM, Cortez D, Weller SK (2010). ATR and ATRIP are recruited to Herpes Simplex Virus type 1 replication compartments even though ATR signaling is disabled..

[30] Wilkinson DE, Weller SK (2004). Recruitment of cellular recombination and repair proteins to sites of herpes simplex virus type 1 DNA replication is dependent on the composition of viral proteins within prereplicative sites and correlates with the induction of the DNA damage response.. J Virol.

[31] Taylor TJ, Knipe DM (2004). Proteomics of herpes simplex virus replication compartments: association of cellular DNA replication, repair, recombination, and chromatin remodeling proteins with ICP8.. J Virol.

[32] Ojala PM, Sodeik B, Ebersold MW, Kutay U, Helenius A (2000). Herpes simplex virus type 1 entry into host cells: reconstitution of capsid binding and uncoating at the nuclear pore complex in vitro.. Mol Cell Biol.

[33] Manders EM, Stap J, Brakenhoff GJ, van Driel R, Aten JA (1992). Dynamics of three-dimensional replication patterns during the S-phase, analysed by double labelling of DNA and confocal microscopy.. J Cell Sci 103 (Pt.

[34] Everett RD, Parada C, Gripon P, Sirma H, Orr A (2008). Replication of ICP0-null mutant herpes simplex virus type 1 is restricted by both PML and Sp100.. J Virol.

[35] Stewart GS, Panier S, Townsend K, Al-Hakim AK, Kolas NK (2009). The RIDDLE syndrome protein mediates a ubiquitin-dependent signaling cascade at sites of DNA damage.. Cell.

[36] Bekker-Jensen S, Lukas C, Melander F, Bartek J, Lukas J (2005). Dynamic assembly and sustained retention of 53BP1 at the sites of DNA damage are controlled by Mdc1/NFBD1.. J Cell Biol.

[37] Lukas J, Lukas C, Bartek J (2004). Mammalian cell cycle checkpoints: signalling pathways and their organization in space and time.. DNA Repair (Amst).

[38] Huen MS, Chen J (2008). The DNA damage response pathways: at the crossroad of protein modifications.. Cell Res.

[39] Rogakou EP, Pilch DR, Orr AH, Ivanova VS, Bonner WM (1998). DNA double-stranded breaks induce histone H2AX phosphorylation on serine 139.. J Biol Chem.

[40] Paull TT, Rogakou EP, Yamazaki V, Kirchgessner CU, Gellert M (2000). A critical role for histone H2AX in recruitment of repair factors to nuclear foci after DNA damage.. Curr Biol.

[41] Celeste A, Fernandez-Capetillo O, Kruhlak MJ, Pilch DR, Staudt DW (2003). Histone H2AX phosphorylation is dispensable for the initial recognition of DNA breaks.. Nat Cell Biol.

[42] Ward IM, Minn K, Jorda KG, Chen J (2003). Accumulation of checkpoint protein 53BP1 at DNA breaks involves its binding to phosphorylated histone H2AX.. J Biol Chem.

[43] Celeste A, Petersen S, Romanienko PJ, Fernandez-Capetillo O, Chen HT (2002). Genomic instability in mice lacking histone H2AX.. Science.

[44] Falck J, Coates J, Jackson SP (2005). Conserved modes of recruitment of ATM, ATR and DNA-PKcs to sites of DNA damage.. Nature.

[45] Lee JH, Paull TT (2005). ATM activation by DNA double-strand breaks through the Mre11-Rad50-Nbs1 complex.. Science.

[46] You Z, Chahwan C, Bailis J, Hunter T, Russell P (2005). ATM activation and its recruitment to damaged DNA require binding to the C terminus of Nbs1.. Mol Cell Biol.

[47] Schultz LB, Chehab NH, Malikzay A, Halazonetis TD (2000). p53 binding protein 1 (53BP1) is an early participant in the cellular response to DNA double-strand breaks.. J Cell Biol.

[48] Huen MS, Grant R, Manke I, Minn K, Yu X (2007). RNF8 transduces the DNA-damage signal via histone ubiquitylation and checkpoint protein assembly.. Cell.

[49] Mailand N, Bekker-Jensen S, Faustrup H, Melander F, Bartek J (2007). RNF8 ubiquitylates histones at DNA double-strand breaks and promotes assembly of repair proteins.. Cell.

[50] Doil C, Mailand N, Bekker-Jensen S, Menard P, Larsen DH (2009). RNF168 binds and amplifies ubiquitin conjugates on damaged chromosomes to allow accumulation of repair proteins.. Cell.

[51] Kolas NK, Chapman JR, Nakada S, Ylanko J, Chahwan R (2007). Orchestration of the DNA-damage response by the RNF8 ubiquitin ligase.. Science.

[52] Galanty Y, Belotserkovskaya R, Coates J, Polo S, Miller KM (2009). Mammalian SUMO E3-ligases PIAS1 and PIAS4 promote responses to DNA double-strand breaks.. Nature.

[53] Morris JR, Boutell C, Keppler M, Densham R, Weekes D (2009). The SUMO modification pathway is involved in the BRCA1 response to genotoxic stress.. Nature.

[54] Wilkinson DE, Weller SK (2003). The role of DNA recombination in herpes simplex virus DNA replication.. IUBMB Life.

[55] Wilkinson DE, Weller SK (2006). Herpes simplex virus type I disrupts the ATR-dependent DNA-damage response during lytic infection.. J Cell Sci.

[56] de Napoles M, Mermoud JE, Wakao R, Tang YA, Endoh M (2004). Polycomb group proteins Ring1A/B link ubiquitylation of histone H2A to heritable gene silencing and X inactivation.. Dev Cell.

[57] Fang J, Chen T, Chadwick B, Li E, Zhang Y (2004). Ring1b-mediated H2A ubiquitination associates with inactive X chromosomes and is involved in initiation of X inactivation.. J Biol Chem.

[58] Wang H, Wang L, Erdjument-Bromage H, Vidal M, Tempst P (2004). Role of histone H2A ubiquitination in Polycomb silencing.. Nature.

[59] Placek BJ, Berger SL (2010). Chromatin dynamics during herpes simplex virus-1 lytic infection.. Biochim Biophys Acta.

[60] Knipe DM, Cliffe A (2008). Chromatin control of herpes simplex virus lytic and latent infection.. Nat Rev Microbiol.

[61] Kutluay SB, Triezenberg SJ (2009). Regulation of histone deposition on the herpes simplex virus type 1 genome during lytic infection.. J Virol.

[62] Oh J, Fraser NW (2008). Temporal association of the herpes simplex virus genome with histone proteins during a lytic infection.. J Virol.

[63] Placek BJ, Huang J, Kent JR, Dorsey J, Rice L (2009). The histone variant H3.3 regulates gene expression during lytic infection with herpes simplex virus type 1.. J Virol.

[64] Lacasse JJ, Schang LM (2010). During lytic infections, herpes simplex virus type 1 DNA is in complexes with the properties of unstable nucleosomes.. J Virol.

[65] Liang Y, Vogel JL, Narayanan A, Peng H, Kristie TM (2009). Inhibition of the histone demethylase LSD1 blocks alpha-herpesvirus lytic replication and reactivation from latency.. Nat Med.

[66] Gu H, Roizman B (2009). Engagement of the lysine-specific demethylase/HDAC1/CoREST/REST complex by herpes simplex virus 1.. J Virol.

[67] Roizman B, Gu H, Mandel G (2005). The first 30 minutes in the life of a virus: unREST in the nucleus.. Cell Cycle.

[68] Carbone R, Pearson M, Minucci S, Pelicci PG (2002). PML NBs associate with the hMre11 complex and p53 at sites of irradiation induced DNA damage.. Oncogene.

[69] Dellaire G, Ching RW, Ahmed K, Jalali F, Tse KC (2006). Promyelocytic leukemia nuclear bodies behave as DNA damage sensors whose response to DNA double-strand breaks is regulated by NBS1 and the kinases ATM, Chk2, and ATR.. J Cell Biol.

[70] Shanbhag NM, Rafalska-Metcalf IU, Balane-Bolivar C, Janicki SM, Greenberg RA (2010). ATM-dependent chromatin changes silence transcription in cis to DNA double-strand breaks.. Cell.

[71] Li L, Halaby MJ, Hakem A, Cardoso R, El Ghamrasni S (2010). Rnf8 deficiency impairs class switch recombination, spermatogenesis, and genomic integrity and predisposes for cancer.. J Exp Med.

[72] Minter-Dykhouse K, Ward I, Huen MS, Chen J, Lou Z (2008). Distinct versus overlapping functions of MDC1 and 53BP1 in DNA damage response and tumorigenesis.. J Cell Biol.

[73] Gripon P, Rumin S, Urban S, Le Seyec J, Glaise D (2002). Infection of a human hepatoma cell line by hepatitis B virus.. Proc Natl Acad Sci U S A.

[74] Ziv Y, Bar-Shira A, Pecker I, Russell P, Jorgensen TJ (1997). Recombinant ATM protein complements the cellular A-T phenotype.. Oncogene.

[75] Carson CT, Schwartz RA, Stracker TH, Lilley CE, Lee DV (2003). The Mre11 complex is required for ATM activation and the G2/M checkpoint.. EMBO J.

[76] Everett RD, Parsy ML, Orr A (2009). Analysis of the functions of herpes simplex virus type 1 regulatory protein ICP0 that are critical for lytic infection and derepression of quiescent viral genomes.. J Virol.

[77] Tiscornia G, Singer O, Verma IM (2006). Production and purification of lentiviral vectors.. Nat Protoc.

[78] Showalter SD, Zweig M, Hampar B (1981). Monoclonal antibodies to herpes simplex virus type 1 proteins, including the immediate-early protein ICP 4.. Infect Immun.

