# Supplementary figures and images for: The Intrinsic Antiviral Defense to Incoming HSV-1 Genomes Includes Specific DNA Repair Proteins and Is Counteracted by the Viral Protein ICP0
Source: PLoS Pathog. 2011 Jun 16;7(6):e1002084. doi: 10.1371/journal.ppat.1002084 (PMC3116817; doi:10.1371/journal.ppat.1002084)

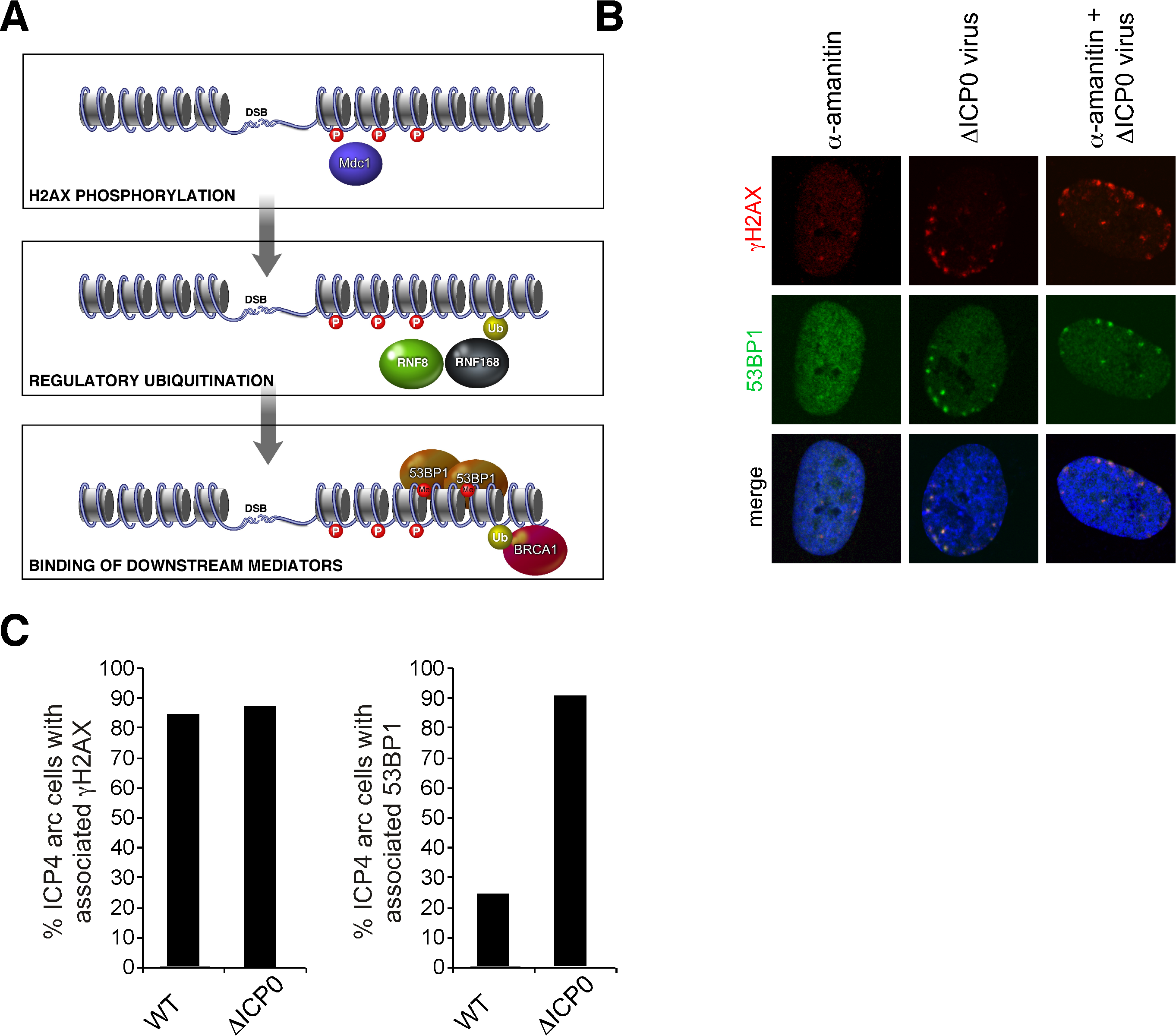

Supplement: Figure S1 — Overview of the cellular response to DNA damage, requirements for recruitment of 53BP1 to sites associated with ICP0-null HSV-1 genomes and quantification of the recruitment phenotype. (A) Simplified overview of the cellular response to DNA damage, indicating the positions of key proteins used in our studies (B) HFF cells were treated with 50 µg/ml α-amanitin for 1 hr prior to infection with ICP0-null HSV-1 at an MOI of 10 for 4 hrs in the presence of 50 µg/ml α-amanitin. Cells were fixed and stained for ICP4 (to confirm that transcription was blocked), and localization of γH2AX and 53BP1 was assessed. Nuclei were stained with DAPI as shown in the merged image. (C) HFF cells were infected with wild-type or ICP0-null HSV-1 at an MOI of 0.001 or 0.1, respectively for 1 hr, and virus was replaced with media containing 1% human serum to limit viral spread. Cells were fixed at 24 hpi, stained for ICP4, and localization of γH2AX (left graph) or 53BP1 (right graph) was assessed in 100 asymmetrically infected cells at edges of plaques. (TIF) [file ppat.1002084.s001.tif]

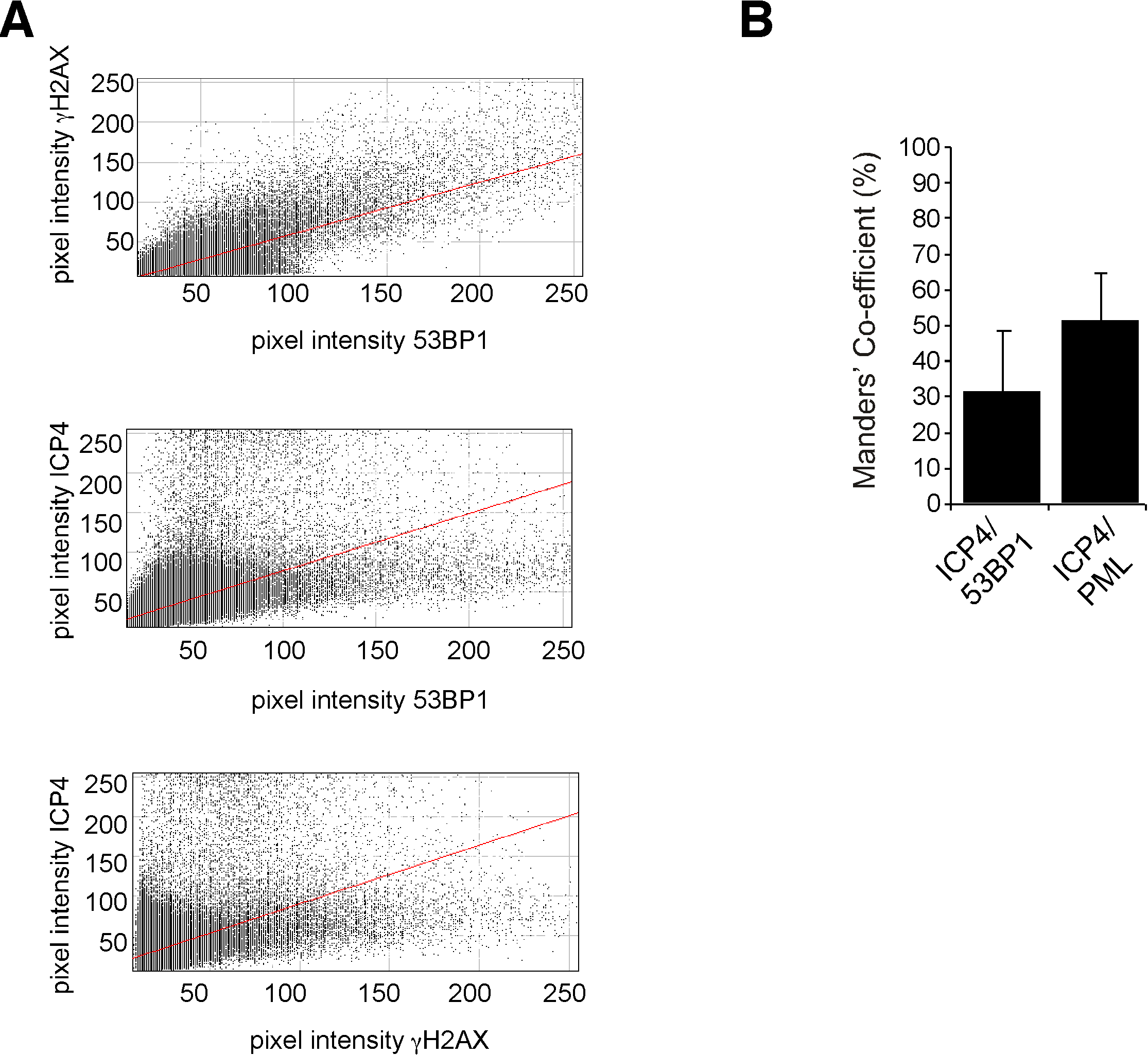

Supplement: Figure S2 — Sites of DNA repair protein accumulation are distinct from viral genomes and de novo ND10 structures. (A) HFF cells were infected with ICP0-null HSV-1 at an MOI of 0.1 for 1 hr, and virus was replaced with media containing 1% human serum. Cells were fixed at 24 hpi, and the correlation between pixels positive for 53BP1 and pixels positive for γH2AX (top panel), 53BP1 and ICP4 (middle panel) or γH2AX and ICP4 (bottom panel) was assessed in one representative asymmetrically infected triple-labeled cell. (B) Cells were infected as in A, and stained to assess ICP4, 53BP1, and PML localization. The Manders' overlap co-efficient was determined for ICP4 and 53BP1 overlap compared to ICP4 and PML overlap in one representative asymmetrically infected triple-labeled cell. (TIF) [file ppat.1002084.s002.tif]

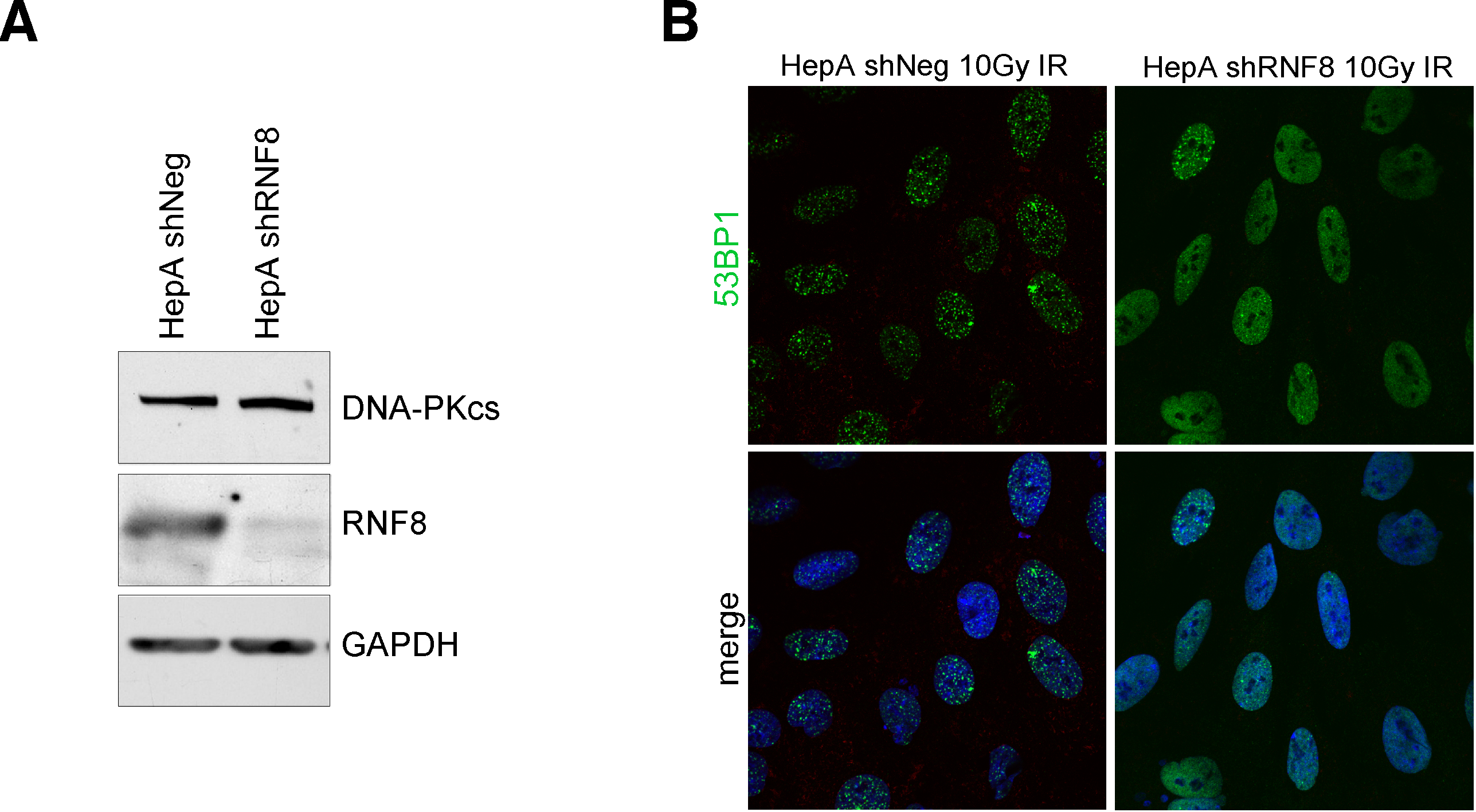

Supplement: Figure S3 — Characterization of RNF8-depleted cells. (A) HepaRG cells were infected with lentivirus expressing shRNA specific to RNF8 as described in the methods section. Cells were isolated under puromycin selection and levels of RNF8 were assessed by western blot. (B) HepaRG shRNF8 cells were irradiated with 10 Gy γIR and the localization of 53BP1 was assessed by immunofluorescence. (TIF) [file ppat.1002084.s003.tif]

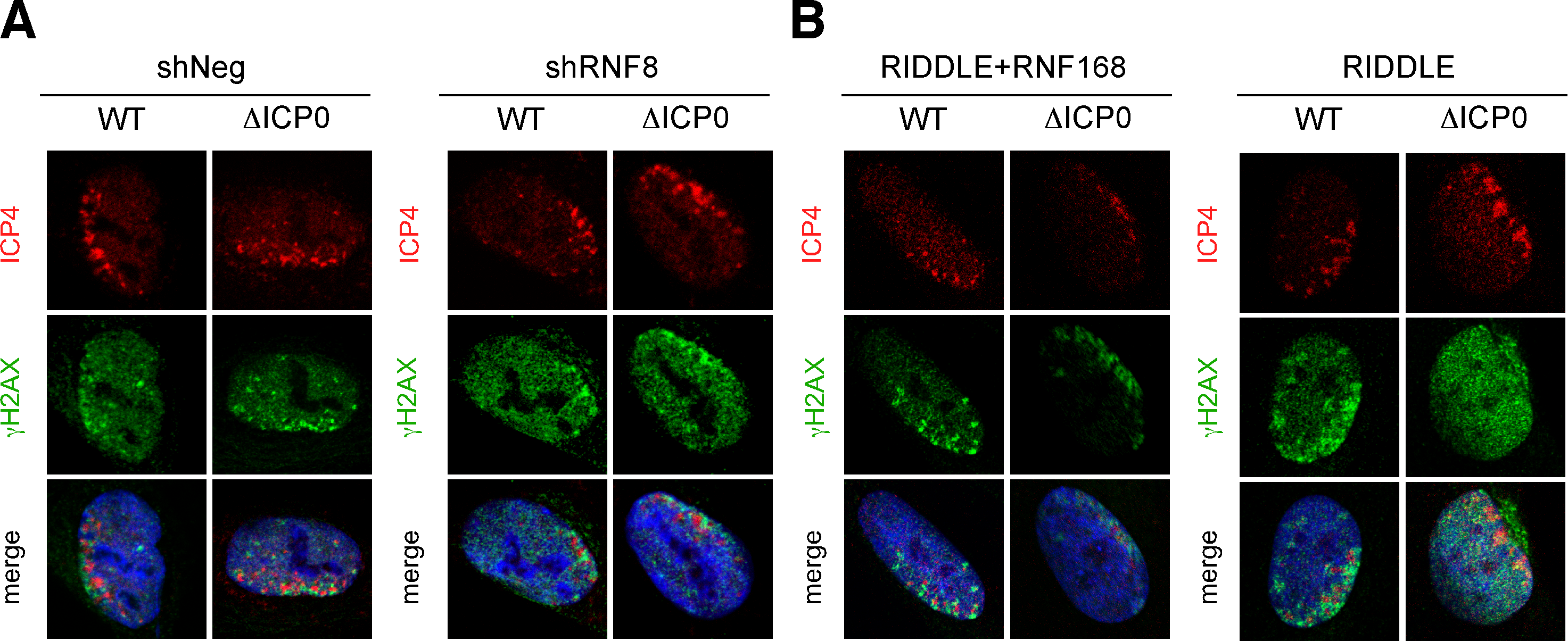

Supplement: Figure S4 — γH2AX accumulation at sites associated with incoming HSV-1 genomes is not dependent on RNF8 or RNF168. (A) Control HepaRG cells or cells in which RNF8 had been depleted using shRNA were infected with wild-type virus at an MOI of 0.001 or ICP0-null HSV-1 at an MOI of 0.1 for 1 hr, and virus was replaced with media containing 1% human serum. Cells were fixed at 24 hpi, stained for ICP4, and γH2AX localization was assessed in asymmetrically infected cells at edges of plaques. (B) RIDDLE cells or RIDDLE cells with HA-tagged RNF168 reconstituted were infected and analyzed as in A. (TIF) [file ppat.1002084.s004.tif]

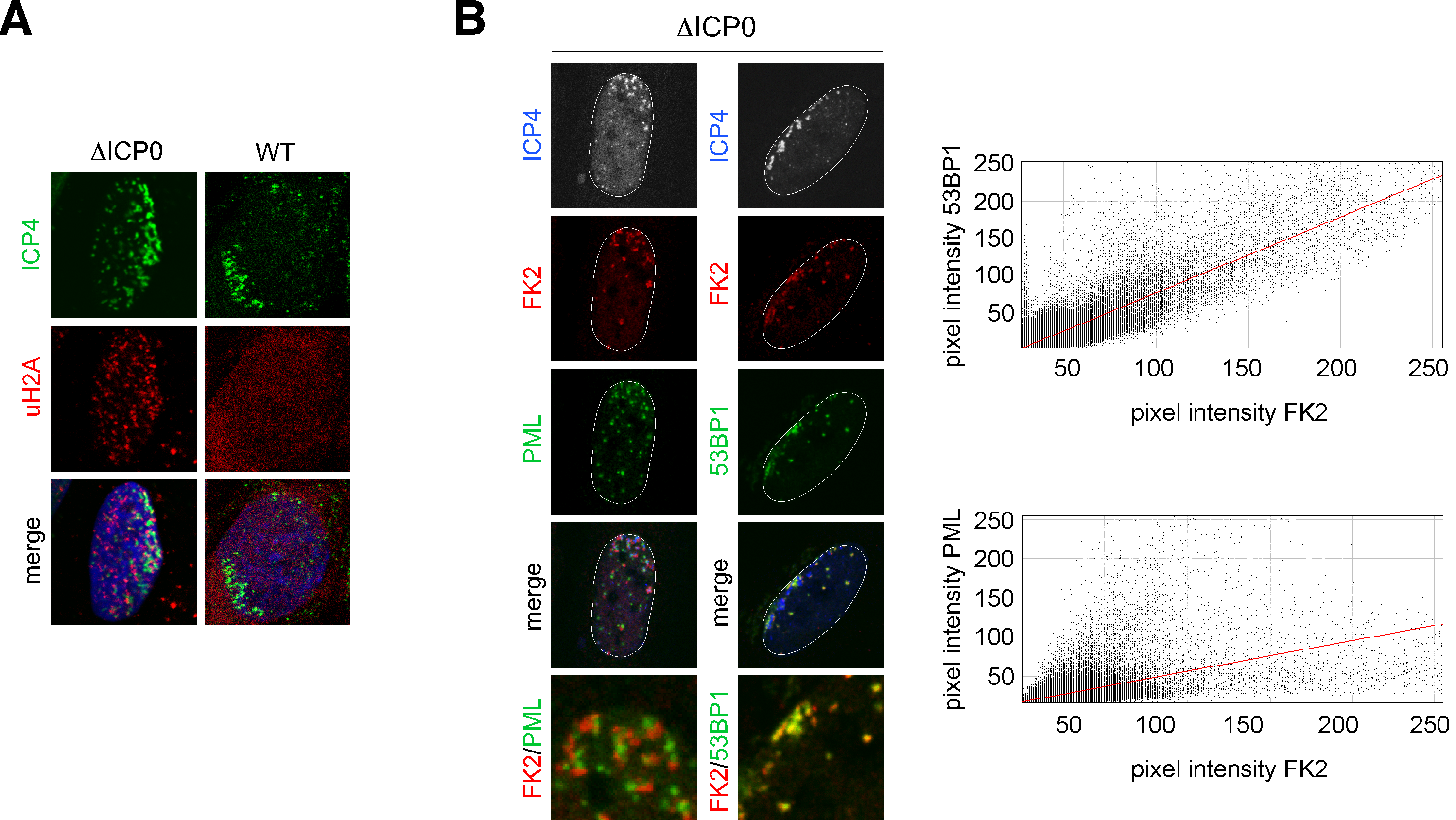

Supplement: Figure S5 — uH2A and conjugated ubiquitin accumulation at sites of incoming ICP0-null HSV-1 genomes. (A) HFF cells were infected with wild-type or ICP0-null HSV-1 at an MOI of 0.001 or 0.1 respectively for 1 hr, and virus was replaced with media containing 1% human serum. Cells were pre-extracted before fixation at 24 hpi, and localization of uH2A was assessed in asymmetrically infected cells at edges of plaques. (B) HFF cells were infected with ICP0-null HSV-1 at an MOI 0.1 for 1 hr, and virus was replaced with media containing 1% human serum. Cells were fixed at 24 hpi, and stained for ICP4, PML, and FK2 (conjugated ubiquitin) or ICP4, 53BP1, and FK2. The correlation between pixels positive for 53BP1 and FK2 (top panel), or PML and ICP4 (bottom panel) was assessed in the images shown. (TIF) [file ppat.1002084.s005.tif]

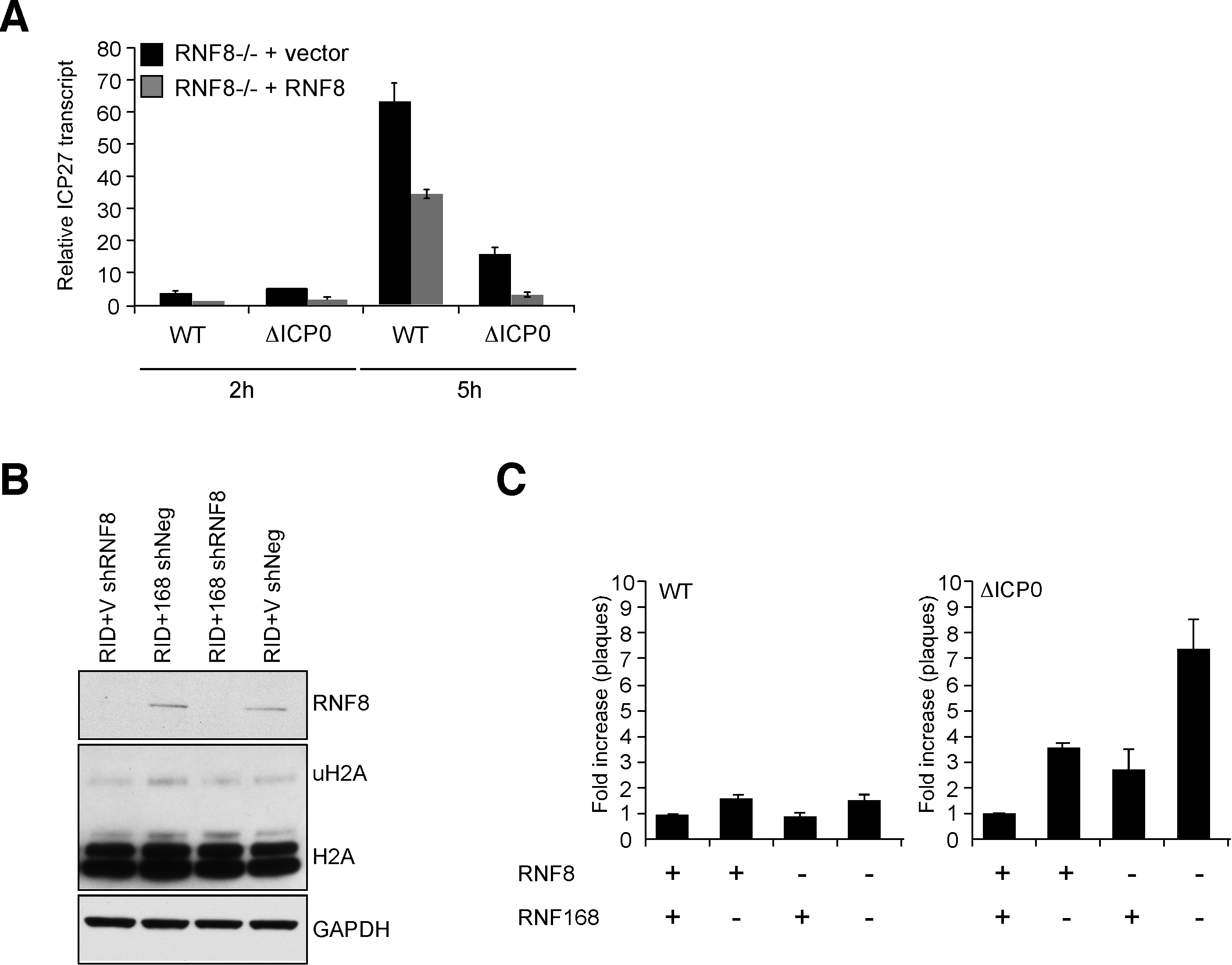

Supplement: Figure S6 — RNF8 represses viral genomes and is part of the intrinsic anti-viral defense. (A) RNF8-/- MEFs transduced with control retrovirus or retrovirus expressing RNF8 were infected with WT or ICP0 null virus at an MOI of 0.01. ICP27 transcripts were detected at 2 or 5 hrs post infection and normalized to a cellular control. In both cell lines, ICP0 null virus was less transcriptionally competent than wild-type, in keeping with the known phenotype of this mutant virus. Experiments were performed in duplicate and averaged. Results are representative of three independent experiments and error is one standard deviation of the duplicate samples. (B) RIDDLE cells complemented either with empty vector or RNF168 were treated with lentivirus expressing either control shRNA or shRNA targeting RNF8. Levels of RNF8 and uH2A were assessed by western blot. (C) Cells depleted for RNF168 and/or RNF8 were infected with wild-type or ICP0-null virus. Relative probabilities of plaque formation were calculated by comparing the numbers of plaques on the different cell lines at each separate dilution of virus. (TIF) [file ppat.1002084.s006.tif]
